# The Fermentation Degree Prediction Model for Tieguanyin Oolong Tea Based on Visual and Sensing Technologies

**DOI:** 10.3390/foods14060983

**Published:** 2025-03-13

**Authors:** Yuyan Huang, Jian Zhao, Chengxu Zheng, Chuanhui Li, Tao Wang, Liangde Xiao, Yongkuai Chen

**Affiliations:** 1 Institute of Digital Agriculture, Fujian Academy of Agricultural Sciences, Fuzhou 350003, China; 18759871027@163.com (Y.H.); zhaojian@faas.cn (J.Z.); dcbazcx@163.com (C.Z.); 18859152710@163.com (C.L.); solow2024@163.com (T.W.); 2 Fujian Zhi Cha Intelligent Technology Co., Anxi 362400, China; 13313872888@163.com

**Keywords:** Tieguanyin oolong tea, weight loss rate, aroma, image features, feature fusion, fermentation

## Abstract

The fermentation of oolong tea is a critical process that determines its quality and flavor. Current fermentation control relies on tea makers’ sensory experience, which is labor-intensive and time-consuming. In this study, using Tieguanyin oolong tea as the research object, features including the tea water loss rate, aroma, image color, and texture were obtained using weight sensors, a tin oxide-type gas sensor, and a visual acquisition system. Support vector regression (SVR), random forest (RF) machine learning, and long short-term memory (LSTM) deep learning algorithms were employed to establish models for assessing the fermentation degree based on both single features and fused multi-source features, respectively. The results showed that in the test set of the fermentation degree models based on single features, the mean absolute error (MAE) ranged from 4.537 to 6.732, the root mean square error (RMSE) ranged from 5.980 to 9.416, and the coefficient of determination (R^2^) values varied between 0.898 and 0.959. In contrast, the data fusion models demonstrated superior performance, with the MAE reduced to 2.232–2.783, the RMSE reduced to 2.693–3.969, and R^2^ increased to 0.982–0.991, confirming that feature fusion enhanced characterization accuracy. Finally, the Sparrow Search Algorithm (SSA) was applied to optimize the data fusion models. After optimization, the models exhibited a MAE ranging from 1.703 to 2.078, a RMSE from 2.258 to 3.230, and R^2^ values between 0.988 and 0.994 on the test set. The application of the SSA further enhanced model accuracy, with the Fusion-SSA-LSTM model demonstrating the best performance. The research results enable online real-time monitoring of the fermentation degree of Tieguanyin oolong tea, which contributes to the automated production of Tieguanyin oolong tea.

## 1. Introduction

Tieguanyin tea is a type of semi-fermented oolong tea that originates from Anxi County, Fujian Province, China [1]. Due to its unique flavor profile, which includes floral notes, a refreshing quality, and a rich and mellow taste, it has become one of the most prevalent oolong teas [2]. The primary processing steps involved in the initial production of Tieguanyin tea include plucking, withering, fermentation (also referred to as ZuoQing, green making, or oxidation), pan firing, shaping, and drying [3,4,5,6]. Mastering the processing of Tieguanyin oolong tea is a profound art, particularly when it comes to fermentation, which is a highly technical process and one of the key steps in creating the distinctive flavor of Tieguanyin tea [7,8].

Fermentation typically involves three to four alternating cycles of tumbling and aeration. Tumbling, accomplished through manual or mechanical means, involves gently shaking the tea leaves and causing them to collide and rub against one another. This action results in slight damage to the leaf edges, facilitating the interaction of enzymes within the leaves with oxygen [9]. Aeration involves spreading the tumbled tea leaves out for a period of time to allow the redistribution of moisture and the release of enzyme substances from damaged cells. Additionally, the exposure of the leaves to air further promotes the fermentation process. Incorrect timing in either tumbling or aeration can lead to over-fermentation or insufficient fermentation, significantly diminishing the quality of the tea [10,11].

Currently, control of the fermentation process mainly relies on the manual sensory experience of tea makers through methods of “touching, smelling, and observing”. “Touching” refers to feeling the softness and hardness of the tea leaves to judge the changes in water content; “smelling” means sniffing the aroma of the tea leaves and perceiving the peak of the characteristic aroma; and “observing” involves monitoring the physical changes on the surface of the tea leaves. During the fermentation process, the edges of tea leaves gradually turn reddish-brown and eventually form the characteristic “green leaves with red edges” [9,12]. However, such methods are subjective and dependent on the individual’s sensory experience, making them labor-intensive and susceptible to external factors such as weather, lighting, and mood. They stand in stark contrast to the standardization, mechanization, and cleanliness demanded by modern agriculture [11,13]. Therefore, it is of great significance to obtain features of tea fermentation in real time, simulate tea makers’ judgment on the degree of fermentation, and achieve online real-time monitoring of the degree of oolong tea fermentation. Currently, based on the judgment and experience of tea makers, single features such as a tea’s water content, aroma, and image have been used to study the fermentation processes of various types of tea.

Moisture content remains one of the most critical parameters in the fermentation process, directly influencing the physical state and chemical reactions of tea, which in turn significantly affect tea quality [14,15]. Chen et al. showed that fresh leaf weight loss rate is the dominant factor affecting aroma changes in tea fermentation [7]. Liu et al. pointed out that the variation pattern of the moisture content in tea leaves during the oolong tea fermentation process is closely related to the quality of the finished tea [16]. Therefore, if changes in moisture content can be measured rapidly, it will be of great significance for guiding the fermentation process.

Tea makers rely on their olfactory experience to judge the degree of fermentation, with the core criterion being the peak appearance of the characteristic aroma of Tieguanyin when the fermentation reaches an optimal level. The essence of this sensory evaluation lies in the dynamic evolution of volatile organic compounds (VOCs). Among these, key aroma components (such as linalool and nerolidol) are produced in large quantities during the fermentation stage, directly influencing sensory perception and the overall quality of tea [8,17,18]. Research has shown that electronic nose technology can effectively simulate human sensory evaluation. For instance, Tseng et al. used an electronic nose to monitor changes in the odor of oolong tea during fermentation and found that the results aligned with the sensory perception trends of tea makers, confirming the feasibility of using electronic noses to replace human sensory evaluation [10]. Han et al. developed a gas sensor detection system capable of effectively distinguishing between four different stages of fermentation in Tieguanyin tea; the prediction accuracy of the BPANN model reached 94.11%, further demonstrating the feasibility of online monitoring of the fermentation process using gas sensors [19].

During fermentation, the polyphenol oxidase in tea leaves reacts with oxygen in the air, leading to changes in color and brightness, which can be effectively captured according to image features [20]. This physical change can be quantitatively captured by machine vision technology, providing an objective alternative to traditional manual observation. Dong et al. demonstrated that the color features of images can quantitatively evaluate the quality indicators and pigment changes during the fermentation process of black tea [21]. Singh et al. classified black tea fermentation via images, achieving an accuracy of 0.895 and a prediction rate of 0.973 in deep learning [22].

The fermentation process of oolong tea involves numerous physicochemical changes [23]. It is difficult to comprehensively and accurately evaluate the fermentation degree of oolong tea by measuring a single characteristic such as moisture, aroma, or image. By fusing data from different sensors, the accuracy of the model can be improved, showing superior performance compared to a single technology. In the case of Longjing tea, Xu et al. utilized an electronic nose and a computer vision system to detect tea aroma and appearance, achieving a maximum classification accuracy of 100% [24,25]. In the research on black tea, Zhou et al. used a computer vision system and an electronic nose to analyze the image and odor characteristic values of black tea, demonstrating that the model effect of multi-source sensors is superior to that of a single sensor, and the classification accuracy of the test set can reach up to 95.6% [26]. However, currently, there is relatively little research on the application of data fusion technology in the real-time monitoring of the oolong tea fermentation process [20].

Therefore, the focus of our research is to simulate the sensory experience of tea makers during actual operations. By collecting multi-source data, such as the water loss rate, aroma, and image changes, we aim to establish a model for the automated and intelligent fermentation process of Tieguanyin tea. This research consists of the following parts: (1) selecting sensors and visual imaging systems capable of online measurement to automatically acquire data on water loss rate, aroma, and image variations during the fermentation of Tieguanyin tea; (2) establishing prediction models of the fermentation degree based on single-factor influences such as water loss rate, aroma, and image features, respectively, as well as models based on data fusion; and (3) comparing the models and further optimizing them using the Sparrow Search Algorithm.

## 2. Materials and Methods

### 2.1. Experimental Design and Sample Selection

Fresh tea leaves were sourced from the Longjuan Township in Anxi County, characterized by a medium-open leaf structure. The tea was harvested on a sunny day between 12:00 and 15:00, with the leaves primarily consisting of one bud and three to four leaves, exhibiting a medium opening. Following harvesting, the fresh leaves underwent sun withering prior to fermentation. The fermentation of Tieguanyin involved three cycles of tumbling and three cycles of aeration, alternating throughout the process. Tumbling was performed using a drum-type tumbling machine with a length of 300 cm and a diameter of 110 cm, at a rotation speed of 22 revolutions per minute. For aeration, the fresh leaves were spread to a thickness of 6 cm on bamboo trays and placed on a drying rack in a fermentation chamber maintained at 21 °C.

Experiments on the fermentation process were conducted in three batches on 28 April, 1 May, and 2 May 2023. The first tumbling lasted 3 min, followed by an aeration period of 50 min; the second tumbling lasted 5 to 7 min, with the subsequent aeration lasting 110 to 140 min; and the third tumbling lasted 12 to 16 min, followed by a 5 to 7 h aeration period. Due to the short intervals between the first two tumbling and aeration cycles, the changes in aroma and appearance features were not significant. Therefore, this study focused on monitoring moisture loss, total aroma, and image data only after the completion of the third tumbling and the commencement of the third aeration. This phase is critical for assessing whether the fermentation level is appropriate [19]. Three tea masters were present on-site to jointly evaluate the fermentation level of the tea. Once the fermentation level was deemed suitable, the process proceeded to the pan-firing stage.

**Figure 1:**
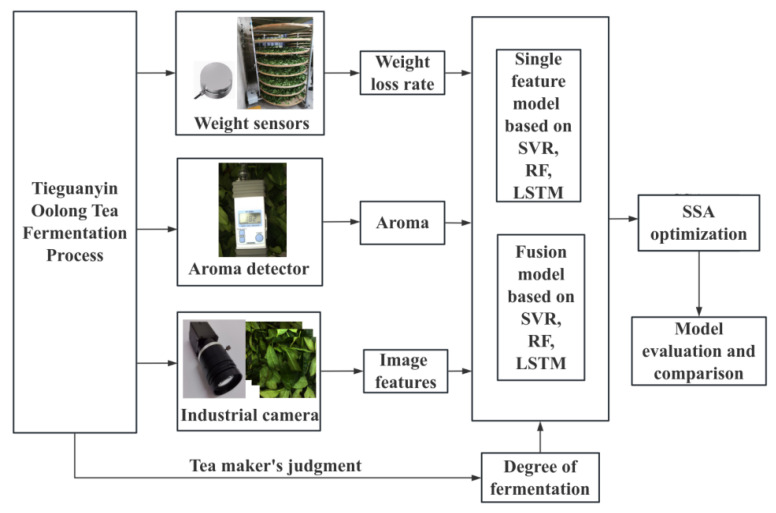
Experimental flowchart.

In the experiment, the fermentation level was quantified on a scale from 0% to 100%, where 0% indicated the beginning of fermentation and 100% signified its completion. Support vector regression (SVR), random forest (RF) machine learning, and long short-term memory (LSTM) deep learning algorithms were employed to develop prediction models for the fermentation level based on both single factors and fused data. The accuracy of these models was compared, and the Sparrow Search Algorithm (SSA) was utilized to further enhance model performance. All model development and performance evaluations were conducted using MATLAB R2023b. Figure 1 presents the experimental flowchart for this study.

### 2.2. Data Collection Equipment

**Figure 2:**
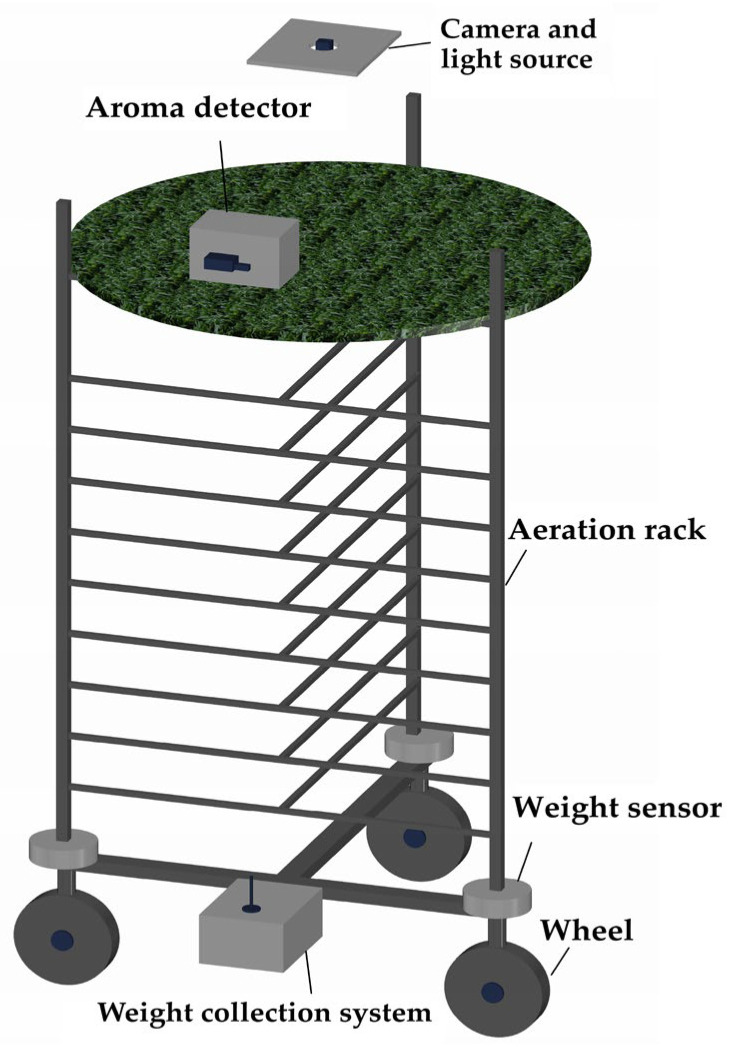
Schematic of the data acquisition equipment.

Figure 2 illustrates a schematic of the data acquisition equipment, which is capable of measuring the water loss rate (WLR), aroma, and image data of the tea leaves. The WLR is measured using three weight sensors, each with a capacity of 0–100 kg, mounted on the three wheels of the aeration rack. These sensors continuously collect the weight of the rack in real time, from which the WLR is calculated. To facilitate mobility, the weight collection system is powered by a lithium battery, and data transmission is accomplished using a RoLa wireless transmission module, which sends the weight data to a host computer for processing and storage. The formula for calculating the WLR is given in Equation (1). In the experiment, three identical racks are employed to independently collect water loss data for the tea on each rack. The aroma data of the tea leaves were collected using a portable odor detector, the XP-329M (COSMOS, Osaka, Japan). The detector, with the built-in high-sensitivity gas sensor (tin oxide type), can measure the total composition of volatile organic compounds present in the tea, which can be used to measure the total aroma value of the tea. During measurements, the instrument was positioned 3 cm above the tea leaves. To minimize measurement errors caused by air movement, the detector was placed in a box measuring 40 cm in length, 30 cm in width, and 25 cm in height, creating a relatively enclosed environment for collecting aroma data. Real-time image data of the tea leaves were captured using three industrial cameras mounted on the ceiling above three aeration racks. The cameras were positioned 25 cm above the tea leaves to monitor the entire fermentation process. The lighting for the photographs was provided by a flat visual light source measuring 20 cm × 20 cm (Juhua Vision Technology Co., Ltd., Dongguan, China). The industrial camera model used was the T-GE1000C-T-CL (Hua Teng Vision, Shenzhen, China), which has a maximum resolution of 3664 × 2748 pixels.

WLR (%) = (W_o_ − W_T_)/(W_o_ − W_S_) × 100% (1)

Here, WLR is the water loss rate of tea leaves, %; W_o_ is the initial mass of tea leaves plus the mass of the aeration rack; W_T_ is the real-time mass of tea leaves plus the mass of the aeration rack; and W_S_ is the mass of the aeration rack. W_S_, W_0_, and W_T_ are all obtained using the weight sensors.

### 2.3. Image Features Extraction

From the original RGB images of the tea leaves, six color feature variables were extracted: red channel average (R), green channel average (G), blue channel average (B), standard deviation of red (R_δ_), standard deviation of green (G_δ_), and standard deviation of blue (B_δ_). The images were then converted from the RGB color space to the HIS color space, from which another six color feature variables were derived: mean hue (H), mean intensity (I), mean saturation (S), standard deviation of hue (H_δ_), standard deviation of intensity (I_δ_), and standard deviation of saturation (S_δ_). Additionally, the RGB images were transformed into grayscale images to extract six texture feature parameters based on a gray-level co-occurrence matrix analysis. These parameters included average gray level (m), standard deviation (δ), smoothness (r), third moment (µ_3_), uniformity (U), and entropy (e) [14,15,27]. The image processing software was developed in Python 3.8 (software copyright number: 2023SR1650911) and was utilized to obtain a total of 18 color and texture features.

### 2.4. Feature Selection via Tree Models

Feature selection using tree models helps identify the most predictive features, effectively reducing data dimensionality, eliminating irrelevant data, and enhancing both the model’s performance and interpretability [28]. The core principle of tree model feature selection is to evaluate the importance of each feature using decision trees or random forest algorithms. During the construction of a decision tree, the algorithm partitions the dataset based on the features in such a way that each resulting subset is as pure as possible. This process allows us to quantify the contribution of each feature, facilitating an assessment of its importance.

### 2.5. Model and Performance Evaluation

#### 2.5.1. SVM

SVM regression aims to identify an optimal hyperplane that best fits the data. By incorporating kernel functions, the SVM can handle both linear and nonlinear relationships, and its strengths lie in its robust generalization capability, as well as its ability to avoid overfitting [29].

#### 2.5.2. RF

RF regression is an ensemble learning method. During the training phase, RF utilizes bootstrap sampling to generate multiple subsets from the original dataset, each of which is used to train an individual decision tree [30]. Ultimately, the predictions from all decision trees are averaged, which can reduce overfitting and improve prediction accuracy [31].

#### 2.5.3. LSTM

LSTM networks are a specialized type of recurrent neural network (RNN). The core of LSTM lies in its three gating mechanisms: the forget gate, input gate, and output gate. By incorporating these gating structures, LSTM networks selectively retain or forget information during the learning process, thereby effectively capturing long-term dependencies [32].

#### 2.5.4. SSA

SSA is an emerging population-based optimization technique. The SSA mimics the dynamic behaviors of sparrow groups and utilizes their unique social hierarchies and behavioral patterns. Through specific computational rules and formulas, the SSA facilitates efficient global and local searches for optimal solutions in complex optimization problems [33].

In a sparrow population, individuals are categorized as discoverers and joiners. Discoverers are responsible for exploring food sources and determining foraging areas and directions, while joiners rely on the information provided by discoverers to locate food. The position update formula for discoverers is defined as follows:

(2) $$X_{i,j}^{t+1}=\left\{\begin{array}{ll} X_{i,j}^{t+1}\cdot exp\left(\frac{-i}{\alpha \cdot iter_{max}}\right) & \;if\;R_{2}<ST\\ X_{i,j}^{t}+Q\cdot L & \;if\;R_{2}\geq ST \end{array}\right.$$

where t is the number of iterations; $X_{i,j}^{t+1}$ is the position information of the i-th sparrow in the j-th dimension during iteration t + 1; α∈(0,1] is a random number; $iter_{max}$ is the maximum number of iterations; R_2_ and ST are warning and safety thresholds, respectively; Q is a random number following a normal distribution; and L is a 1 × d matrix, where each element is −1.

For joiners, the position update formula is the following:

(3) $$X_{i,j}^{t+1}=\left\{\begin{array}{lll} & Q\cdot exp\left(\frac{X_{worst}^{t}-X_{i,j}^{t}}{\alpha \cdot iter_{max}}\right) & \;if\;i<n/2\\ & X_{P}^{t+1}+\left|X_{i,j}^{t}-X_{P}^{t+1}\right|\cdot A^{+}\cdot L & \;otherwise \end{array}\right.$$

where $X_{P}$ is the optimal position currently occupied by the discoverer and $X_{worst}$ is the worst current global position. A is a 1 × d matrix, where each element is randomly assigned a value of 1 or −1; $A^{+}=$ $A^{T}{\left(AA^{T}\right)}^{-1}$. When i > n/2, it indicates that the i-th joiner with lower fitness values cannot obtain food and needs to search elsewhere for food sources.

When the population detects danger, sparrows will adopt anti-predator measures. In this case, the position update formula is given by

(4) $$X_{i,j}^{t+1}=\left\{\begin{array}{ll} X_{best}^{t}+\beta \cdot \left|X_{i,j}^{t}-X_{best}^{t}\right| & if\;f_{i}>f_{g}\\ X_{i,j}^{t}+K\cdot \left(\frac{\left|X_{i,j}^{t}-X_{worst}^{t}\right|}{\left(f_{i}-f_{w}\right)+\varepsilon }\right) & if\;f_{i}=f_{g} \end{array}\right.$$

where $X_{best}$ is the current global optimal position; β is a step length control parameter, which is a random number following a normal distribution with a mean of 0 and a variance of 1; $K\in \left[-1,1\right]$ is a random number; $f_{i}$, $f_{g}$, and $f_{w}$ are the fitness values of the current individual sparrow, the global best and the global worst, respectively; and ε is a minimum constant.

#### 2.5.5. Model Evaluation

The mean absolute error (MAE), root mean square error (RMSE), and coefficient of determination (R^2^) are used to evaluate the regression prediction results. These metrics are defined by Equations (5)–(7). Smaller values of the MAE and RMSE indicate higher predictive accuracy, while a value of R^2^ closer to 1 indicates a stronger explanatory capability of the model [34].

(5) $$MAE=\frac{1}{m}\sum_{i=1}^{m}|y_{i}-{\hat{y}}_{i}|$$

(6) $$RMSE=\sqrt{\frac{1}{m}\sum_{i=1}^{m}{\left(y_{i}-{\hat{y}}_{i}\right)}^{2}}$$

(7) $$R^{2}=1-\frac{\sum_{i=1}^{m}{\left(y_{i}-{\hat{y}}_{i}\right)}^{2}}{\sum_{i=1}^{m}{\left(y_{i}-{\overset{‾}{y}}_{i}\right)}^{2}}$$

Here, $y_{i}$ is the true value, ${\hat{y}}_{i}$ is the predicted value, and m is the number of samples.

## 3. Results and Discussion

### 3.1. Data Acquisition

Aroma data were recorded every 5 min. The industrial cameras captured one image of the tea leaves every minute, resulting in a total of fifteen images collected over 5 min from three cameras. The average image features from these images were utilized as the final dataset. Similarly, WLR data were also recorded every 5 min, and the average WLR derived from the three racks served as the final WLR. Therefore, characteristic data such as the WLR, aroma, and image of tea leaves were obtained for the tea fermentation process, resulting in a total of 220 sets of data collected over three days. The data were randomly divided into training and test sets in a 7:3 ratio, yielding 154 sets of data for training and 66 for testing. The training set was employed for the initial learning and parameter tuning of the model, while the test set was used to evaluate the model’s performance on new data. Given the varied dimensionality of different feature data, Z-score normalization was conducted prior to modeling for each dataset.

### 3.2. Fermentation Degree Model Based on WLR Data

**Figure 3:**
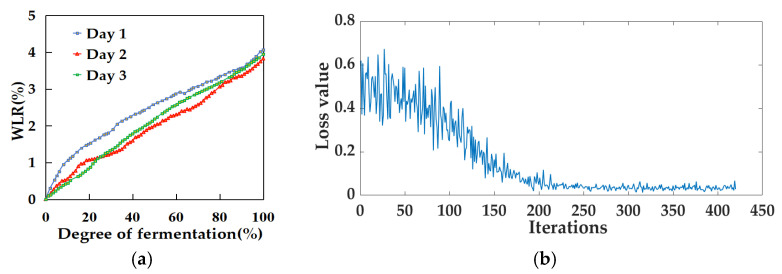
Fermentation degree and modeling monitoring based on WLR data: (**a**) scatter plot of WLR and fermentation degree and (**b**) the loss curve of the WLR-LSTM model.

WLR data were collected over a three-day fermentation period. Based on the collected data, a scatter plot illustrating the relationship between WLR and fermentation degree was generated, as shown in Figure 3a. Observations indicate a gradual increase in the WLR throughout the fermentation process. Specifically, the total WLR was 4.064% on the first day, 3.827% on the second day, and 3.950% on the third day, resulting in an average total WLR of 3.947% for the three days.

**Figure 4:**
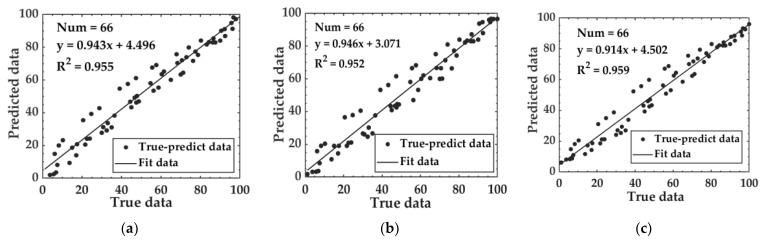
Relationship between predicted data and true data of the test set based on WLR data: (**a**) WLR-SVM; (**b**) WLR-RF; and (**c**) WLR-LSTM.

Using SVR, RF machine learning, and LSTM deep learning algorithms, prediction models for WLR and fermentation progression were developed. To observe the training progression and convergence of the WLR-LSTM model during training, the model’s loss curve was plotted, as shown in Figure 3b. In deep learning, the model loss curve is an intuitive way to measure the difference between the model’s prediction results and the real results. As illustrated in Figure 3b, in the initial training stage of the WLR-LSTM model, the loss value was relatively high, which indicates that there was a large deviation between the model’s prediction results and the actual values in the initial stage. As the number of iterations gradually increased, the model continuously adjusted its own parameters and learned the patterns and rules in the training data, and the loss value gradually decreased accordingly. When the number of iterations reached 220, the loss value basically stabilized and there was no obvious change anymore, which means that the model reached the convergence state. At this moment, the model learned the features of the data to a certain extent and was able to make relatively stable predictions. The performance of models based on water loss rate is presented in Table 1. The MAE for the three models on the test set ranged from 4.537 to 4.986, the RMSE fell between 5.980 and 6.463, and the R^2^ values were between 0.952 and 0.959, indicating that the performance of the models was comparable. Figure 4 shows the relationship between the predicted data and the true data of the test set based on the WLR data.

### 3.3. Fermentation Degree Model Based on Aroma Data

**Figure 5:**
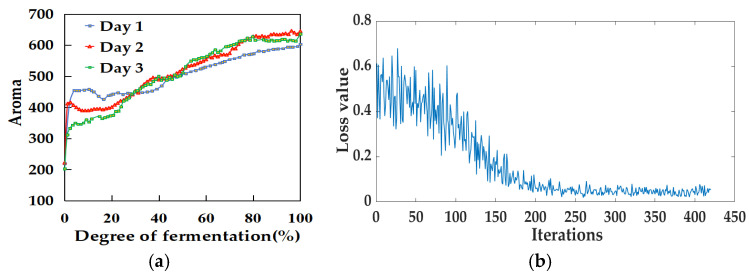
Fermentation degree and modeling monitoring based on aroma data: (**a**) scatter plot of aroma and fermentation degree and (**b**) the loss curve of the Aroma-LSTM model.

Using the aroma data collected over three days, a scatter plot illustrating the relationship between aroma and the fermentation degree was generated, as shown in Figure 5a. The aroma of the tea leaves showed a sharp increase at the beginning of the data collection, primarily due to the unstable collection environment. Subsequently, the aroma tended to stabilize and exhibited a gradual upward trend. The maximum aroma value recorded on the first day, indicating the end of fermentation, was 603; on the second day, it was 645; and on the third day, it was 636, resulting in an average maximum aroma value of 628 over the three days.

**Figure 6:**
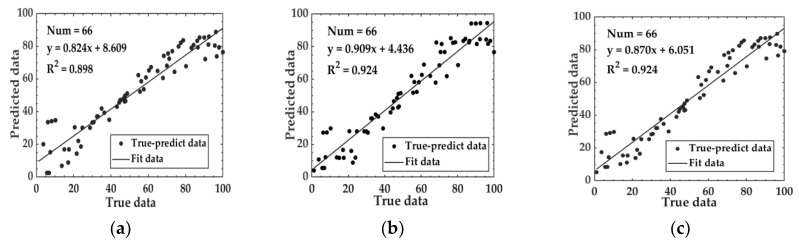
Relationship between predicted data and true data of the test set based on aroma data: (**a**) Aroma-SVM; (**b**) Aroma-RF; and (**c**) Aroma-LSTM.

Machine learning algorithms such as SVR and RF, along with the LSTM deep learning algorithm, were utilized to establish a prediction model for the relationship between aroma and the degree of fermentation. The loss curve of the Aroma-LSTM model is depicted in Figure 5b. The model converged after approximately 200 iterations. By this time, the loss value calculated by the loss function had stabilized, and the model could already make relatively stable predictions. The performance of models based on aroma data is shown in Table 2. The MAE for the three models on the test set ranged from 6.015 to 6.732, the RMSE varied between 8.109 and 9.416, and the R^2^ values fell within the range of 0.898 to 0.924. Notably, the Aroma-RF and Aroma-LSTM models demonstrated better performance compared with the Aroma-SVM model. The relationship between the predicted data and the true data of the test set based on aroma data is illustrated in Figure 6.

### 3.4. Fermentation Degree Prediction Model Based on Image Features

#### 3.4.1. Changes in Surface Color During Fermentation

**Figure 7:**
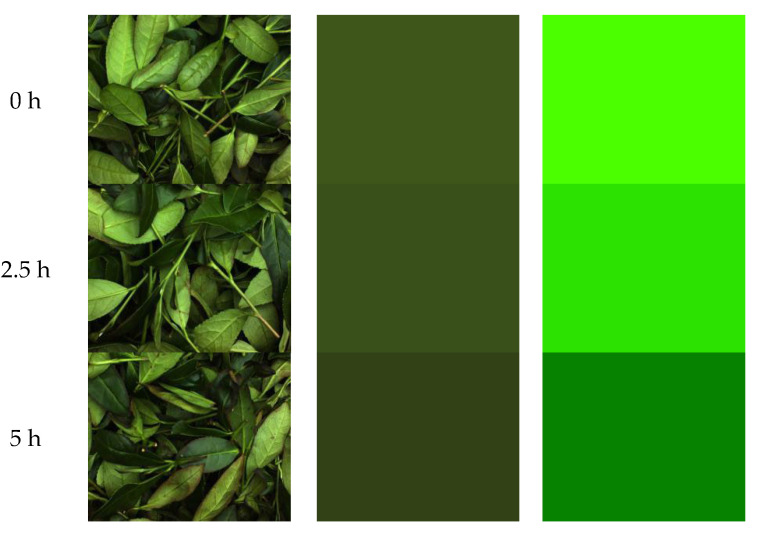

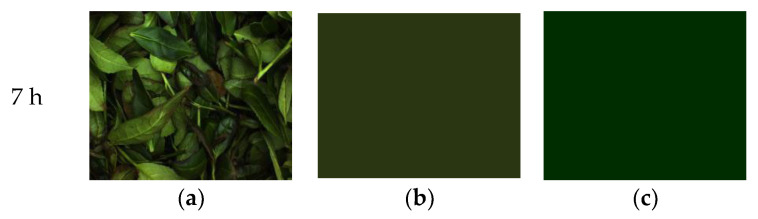
Surface color change during fermentation: (**a**) original image; (**b**) average color image; and (**c**) enhanced average color image.

To reflect the changes in surface color during the fermentation of tea leaves more intuitively, one original image was selected from various time points, and the average color image for that time point was extracted, as shown in Figure 7a,b. To enhance the visibility of differences, we increased the saturation, brightness, and contrast to better show the color variations throughout the fermentation process, as depicted in Figure 7c. As can be seen from Figure 7c, the images exhibited noticeable differences in color and brightness across different stages of fermentation. As time progressed, the edges of the leaves turned red, and the overall color of the leaves changed from an initial bright green to darker green, with some leaves even displaying traces of reddish-brown. This phenomenon is consistent with the typical color changes observed in tea leaves during fermentation. These changes are attributed to the oxidation of polyphenolic compounds in the tea leaves, forming substances such as theaflavins, thearubigins, and theabrownins [20].

#### 3.4.2. Feature Selection Results from Tree Models Based on Image Features

**Figure 8:**
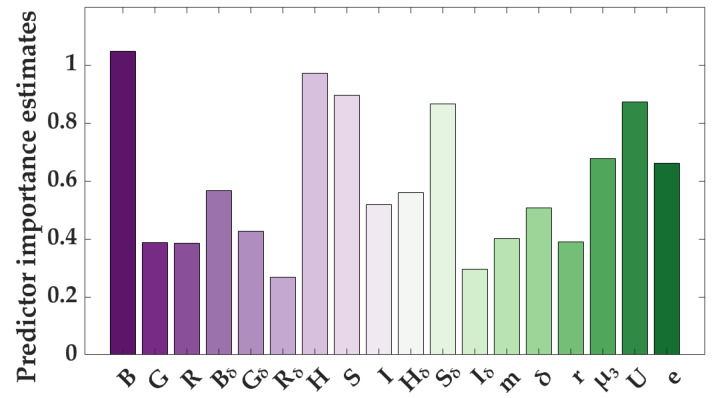
The results of the feature selection for image features.

For feature selection, a random forest was employed to construct a supervised model, allowing for the assessment of feature importance during the training process. The results of the feature selection for image features are illustrated in Figure 8, where the *x*-axis represents each feature, and the *y*-axis represents the estimated importance value. A higher value indicates a greater influence on the output and greater importance of the feature. The importance scores for the image features are as follows: B = 1.047, H = 0.970, S = 0.895, U = 0.872, S_δ_ = 0.865, µ_3_ = 0.678, e = 0.662, B_δ_ = 0.567, H_δ_ = 0.560, I = 0.518, δ = 0.507, G_δ_ = 0.426, m = 0.400, r = 0.390, G = 0.387, R = 0.384, I_δ_ = 0.296, and R_δ_ = 0.269. From these importance metrics, it is evident that the blue channel average (B), mean hue (H), and mean saturation (S) are the three most critical features among the image characteristics. During the feature selection process, a threshold of 0.5 was set based on the model’s testing performance, which meant that only features with an importance score exceeding this threshold were selected. In total, 11 image features were chosen for modeling.

#### 3.4.3. Model Results Based on Image Features

**Figure 9:**
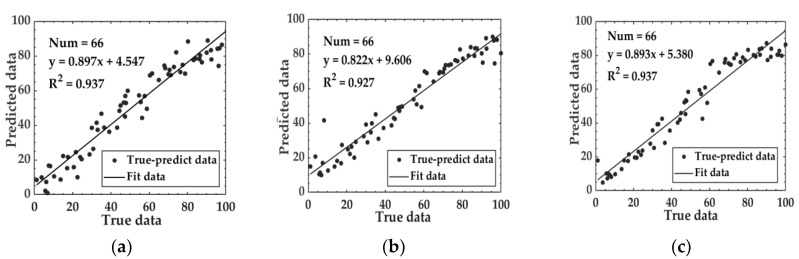
The relationship between predicted data and true data of the test set based on the image features data: (**a**) Image-SVM; (**b**) Image-RF; and (**c**) Image-LSTM.

**Figure 10:**
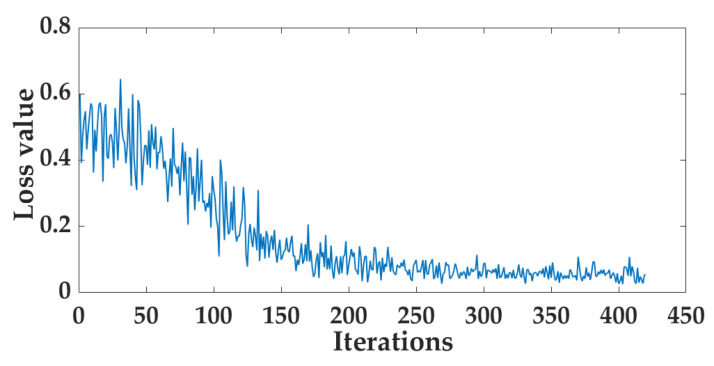
The loss curve of the Image-LSTM model.

Predictive models for the relationship between image features and fermentation progress were developed using machine learning algorithms including SVR, RF, and LSTM. The performance metrics of these models are listed in Table 3. The testing results of the three models were similar, with MAE values ranging from 5.378 to 6.211, RMSE values between 7.392 and 7.953, and R^2^ values ranging from 0.927 to 0.937. The relationship between predicted data and true data of the test set based on the image features data is depicted in Figure 9. Additionally, the loss function curve of the Image-LSTM model, varying with the number of iterations, is shown in Figure 10. The model converged after approximately 230 iterations. At this time, the loss value calculated by the loss function tended to be stable, and the model could already make relatively stable predictions.

### 3.5. Fermentation Degree Prediction Model Based on Fusion Feature Data

#### 3.5.1. Feature Selection Results from Tree Models Based on Fusion Feature Data

**Figure 11:**
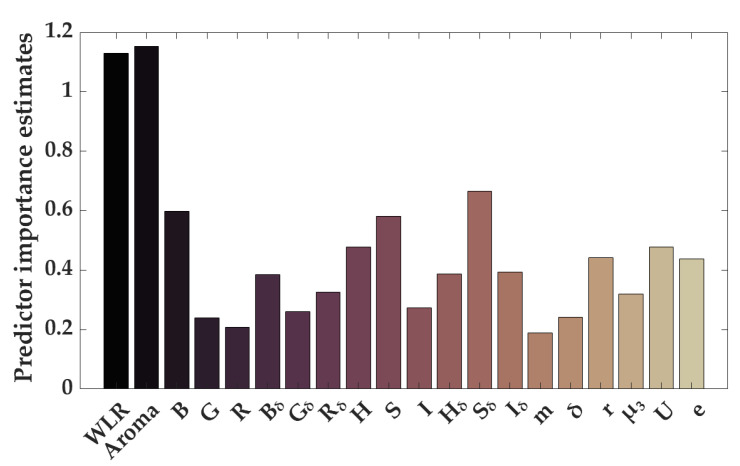
Feature selection results from tree model based on fusion feature data.

Feature selection results from the tree models were performed using fusion feature data, including the WLR, aroma, and image features. The results are shown in Figure 11. The features are ranked according to their importance from highest to lowest as follows: Aroma = 1.153, WLR = 1.130, S_δ_ = 0.665, B = 0.599, S = 0.581, U = 0.478, H = 0.477, r = 0.441, e = 0.438, I_δ_ = 0.394, H_δ_ = 0.386, B_δ_ = 0.385, R_δ_ = 0.326, µ_3_ = 0.320, I = 0.275, G_δ_ = 0.259, δ = 0.242, G = 0.239, R = 0.208, and m = 0.198. The results indicate that the aroma, WLR, standard deviation of saturation (S_δ_), blue channel average (B), and mean saturation (S) are the five most important features. Based on the model testing performance, a threshold of 0.4 was established for feature selection, meaning that only features with scores exceeding this threshold were included. In total, nine features were selected for modeling.

#### 3.5.2. Model Results Based on Fusion Feature Data

**Figure 12:**
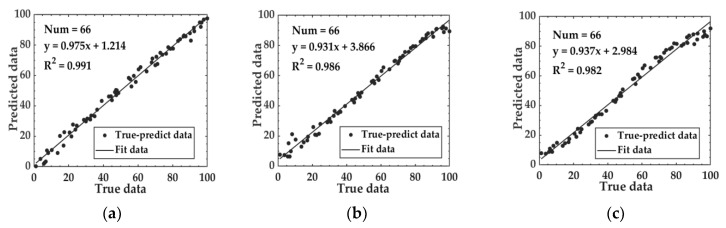
The relationship between predicted data and true data based on fusion feature data: (**a**) Fusion-SVM; (**b**) Fusion-RF; and (**c**) Fusion-LSTM.

We utilized SVR, RF, and LSTM algorithms to develop predictive models for the relationship between fusion feature data and the fermentation degree. The performance of these models is reported in Table 4, yielding a MAE of 2.232–2.783, RMSE of 2.693–3.969, and R^2^ values of 0.982–0.991 on the test set. The relationship between predicted data and true data of the test set based on fusion feature data is depicted in Figure 12.

### 3.6. Model Comparison

**Figure 13:**
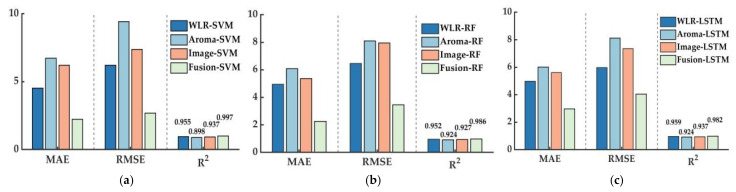
Comparative effect of models in different datasets: (**a**) SVR; (**b**) RF; and (**c**) LSTM.

Figure 13 illustrates the modeling performance of the SVR, RF, and LSTM models across different datasets. For the SVR models, including WLR-SVR, Aroma-SVR, Image-SVR, and Fusion-SVR, the MAE values were 4.537, 6.732, 6.211, and 2.232, respectively. The RMSE values were 6.214, 9.416, 7.392, and 2.693, and the R^2^ values were 0.955, 0.898, 0.937, and 0.991, respectively. In the RF models, which include WLR-RF, Aroma-RF, Image-RF, and Fusion-RF, the MAE values were 4.956, 6.082, 5.378, and 2.249, respectively; the RMSE values were 6.463, 8.109, 7.953, and 3.447; and the R2 values were 0.952, 0.924, 0.927, and 0.986. For the LSTM models—WLR-LSTM, Aroma-LSTM, Image-LSTM, and Fusion-LSTM—the MAE values were 4.986, 6.015, 5.675, and 2.783, respectively; the RMSE values were 5.980, 8.123, 7.408, and 3.969; and the R^2^ values were 0.959, 0.924, 0.937, and 0.982. The results indicate that compared to models based on single feature data, models based on fusion feature data can significantly reduce MAE and RMSE values while increasing the R^2^ value, demonstrating a notable improvement in model accuracy.

### 3.7. Model Optimization

**Figure 14:**
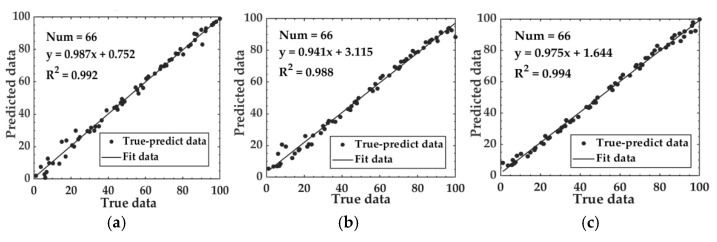
Relationship between predicted data and true data based on fusion feature data: (**a**) Fusion-SSA-SVM; (**b**) Fusion-SSA-RF; and (**c**) Fusion-SSA-LSTM.

Based on fusion feature data, the Sparrow Search Algorithm (SSA) was applied to enhance the SVR, RF, and LSTM models, thereby establishing predictive models for the relationship between fused features and the fermentation degree. The performance metrics of these models are detailed in Table 5, and the relationship between the predicted data and true data is illustrated in Figure 14. For the Fusion-SSA-SVR model, key optimizations included the kernel function scaling factor set to KernelScale = 2.449, the upper limit of sample weights adjusted to BoxConstraint = 19.785, and the sensitivity band width set to Epsilon = 0.001. The Fusion-SSA-RF model optimized the number of decision trees to Tree num = 68 and the minimum leaf node size to MinLeafSize = 1. For the Fusion-SSA-LSTM model, the number of hidden units in the two LSTM layers was optimized, with the first LSTM layer containing 76 hidden units and the second layer containing 47 hidden units. Additionally, the dropout rate was optimized to 0.258.

**Figure 15:**
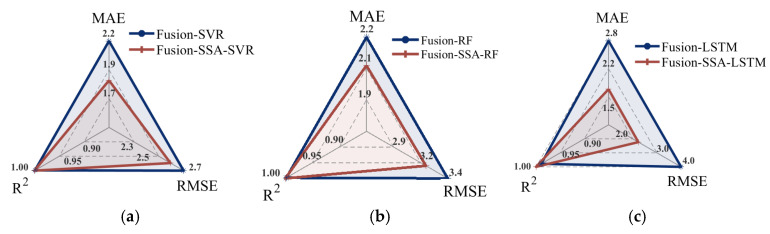
The radar chart comparing model performance before and after SSA optimization: (**a**) SVR; (**b**) RF; and (**c**) LSTM.

**Figure 16:**
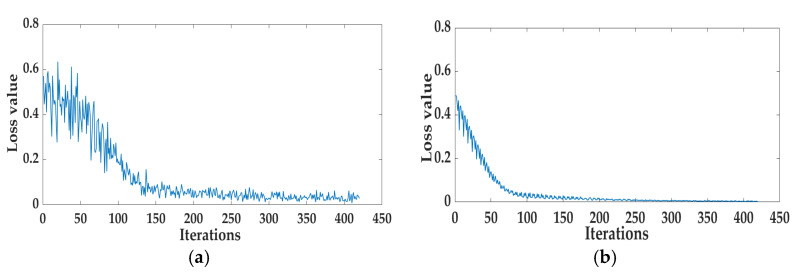
Model loss curve chart: (**a**) Fusion-LSTM and (**b**) Fusion-SSA-LSTM.

The comparison between the Fusion-SVM model and the Fusion-SSA-SVM model reveals a notable improvement in the test set, with the MAE decreasing from 2.232 to 1.834, the RMSE declining from 2.693 to 2.600, and the R^2^ value increasing from 0.991 to 0.992. Similar trends were also observed in the comparison between the Fusion-RF model and the Fusion-SSA-RF model, with the MAE decreasing from 2.249 to 2.078, the RMSE dropping from 3.969 to 3.230, and the R^2^ value rising from 0.986 to 0.988. For the Fusion-LSTM model compared with the Fusion-SSA-LSTM model, the MAE dropped from 2.786 to 1.703, the RMSE declined from 3.939 to 2.258, and the R^2^ value increased from 0.982 to 0.994. The radar chart comparing model performance before and after SSA optimization is presented in Figure 15. The results indicate that the SSA consistently enhanced model accuracy, with the Fusion-SSA-LSTM model demonstrating the best performance on the test set and achieving the highest simulation effectiveness. The loss function curves for the Fusion-LSTM and Fusion-SSA-LSTM models are presented in Figure 16. As can be seen from Figure 16a, for the Fusion-LSTM model, when the number of iterations reached 150, the loss value basically stabilized and there was no obvious change anymore, indicating that the model had reached the convergence state. From Figure 16b, it can be observed that the Fusion-SSA-LSTM model performed even better. The model reached the convergence state after only 100 iterations. Therefore, the Fusion-SSA-LSTM model optimized by the SSA is able to learn the features in the data more efficiently, thus accelerating the decrease in the loss value. It has a significant advantage in terms of convergence speed, which is of great significance for improving the training efficiency of the model and its application effects.

In order to test the running speed of the Fusion-SSA-LSTM model, five rounds of training and testing were carried out on an experimental computer with an Intel(R) Core(TM) i5-10400F CPU @ 2.90 GHz, an NVIDIA GeForce GTX 1660 SUPER graphics card, and 16 GB of memory. After the model was generated, a new set of feature data was predicted. The results show that the average time taken to complete one round of training and testing is 101.64 s, and the average time taken to complete the prediction of one set of new data is 0.012 s. Therefore, this running speed is sufficient to meet the system requirements for real-time monitoring of the tea fermentation production line.

## 4. Conclusions

Fermentation is one of the key processes in Tieguanyin oolong tea production, significantly influencing its flavor and aroma. Therefore, there is an urgent need for rapid assessment of the fermentation degree in the industrial production of Tieguanyin tea. This study focuses on modeling the fermentation process of Tieguanyin tea, aiming to enhance the intelligence of fermentation assessment through the fusion of multi-source information, including water loss rate (WLR), aroma, and image features. The results indicate that when considering multi-source information, such as the WLR, aroma, and variations in image features, the proposed approach offers several advantages over single-feature monitoring methods. These advantages include a large amount of information, strong comprehensive ability, high fault tolerance, and similarity to human cognitive processes, all of which contribute to increased model accuracy. Additionally, the introduction of the Sparrow Search Algorithm (SSA) further enhances the model’s accuracy and convergence speed, with the Fusion-SSA-LSTM model achieving a R^2^ value of 0.994 on the prediction set. This model not only enables precise control over the fermentation process but also significantly improves the quality of the tea, providing a scientific basis for the production of Tieguanyin oolong tea.

## Figures and Tables

**Table 1:** Performance of models based on WLR data.

Model	Training Set			Test Set		
	MAE	RMSE	R^2^	MAE	RMSE	R^2^
WLR-SVM	4.056	5.691	0.961	4.537	6.214	0.955
WLR-RF	3.430	4.595	0.975	4.956	6.463	0.952
WLR-LSTM	4.552	5.624	0.962	4.986	5.980	0.959

**Table 2:** Performance of models based on aroma data.

Model	Training Set			Test Set		
	MAE	RMSE	R^2^	MAE	RMSE	R^2^
Aroma-SVM	6.643	9.485	0.892	6.732	9.416	0.898
Aroma-RF	4.368	5.688	0.961	6.082	8.109	0.924
Aroma-LSTM	5.662	7.097	0.940	6.015	8.123	0.924

**Table 3:** Performance of models based on image features.

Model	Training Set			Test Set		
	MAE	RMSE	R^2^	MAE	RMSE	R^2^
Image-SVM	5.815	7.621	0.930	6.211	7.392	0.937
Image-RF	3.829	5.55	0.963	5.378	7.953	0.927
Image-LSTM	5.621	7.441	0.934	5.675	7.408	0.937

**Table 4:** Performance of models based on fusion feature data.

Model	Training Set			Test Set		
	MAE	RMSE	R^2^	MAE	RMSE	R^2^
Fusion-SVM	2.835	3.635	0.984	2.232	2.693	0.991
Fusion-RF	1.582	2.335	0.993	2.249	3.447	0.986
Fusion-LSTM	2.517	3.668	0.984	2.783	3.969	0.982

**Table 5:** Performance of optimization models based on fusion feature data.

Model	Training Set			Test Set		
	MAE	RMSE	R^2^	MAE	RMSE	R^2^
Fusion-SSA-SVM	2.335	3.775	0.985	1.834	2.600	0.992
Fusion-SSA-RF	0.958	1.386	0.998	2.078	3.230	0.988
Fusion-SSA-LSTM	1.689	2.257	0.994	1.703	2.258	0.994

## Data Availability

The original contributions presented in the study are included in the article; further inquiries can be directed at the corresponding author.
